# Decline of Orientation and Direction Sensitivity in the Aging Population

**DOI:** 10.3389/fnins.2021.643414

**Published:** 2021-04-07

**Authors:** Lin Xia, He Chen, Jiong Dong, Sha Luo, Lixia Feng

**Affiliations:** ^1^Department of Ophthalmology, First Affiliated Hospital of Anhui Medical University, Hefei, China; ^2^Anhui Branch of National Clinical Research Center for Ocular Diseases, Hefei, China; ^3^Hefei National Laboratory for Physical Sciences at Microscale, School of Life Sciences, University of Science and Technology of China, Hefei, China

**Keywords:** vision, orientation, motion direction, aging, perception deficits, psychometric function

## Abstract

While the aging population is growing, our knowledge regarding age-related deterioration of visual perception remains limited. In the present study, we investigated the effects of aging on orientation and direction sensitivity in a healthy population using a weighted up–down adaptive method to improve the efficiency and reliability of the task. A total of 57 healthy participants aged 22–72 years were included and divided into old and young groups. Raw experimental data were processed using a psychometric method to determine the differences between the two groups. In the orientation task, the threshold of the discrimination angle and bias (i.e., the difference between the perceived midpoint from the logistic function and the reference point) was increased, while the lapsing rate (i.e., 1—the maximum logistic function) did not significantly change in the old group compared with the young group. In the motion direction task, the threshold, bias, and lapsing rate were significantly increased in the old group compared with the young group. These results suggest that the decreased ability of old participants in discrimination of stimulus orientation and motion direction could be related to the impaired function of visual cortex.

## Introduction

Vision and other sensory organs deteriorate with aging, thereby decreasing the quality of life of the aging population (Andersen, 2012). Due to ocular changes, lens opacification and reduced ocular size limit light transmittance and increase scattering (van de Kraats and van Norren, 2007; Petrash, 2013; Guillon et al., 2016), resulting in impaired quality of light projected to the retina (Weale, 1988; IJspeert et al., 1990). However, decreased optical quality does not account for all aging-associated changes in the visual sensory system; microstructural changes and aging central nervous system also play a role in visual sensory dysfunction. For example, certain photoreceptor and retina ganglion cells are lost during aging (Panda-Jonas et al., 1995; Harman et al., 2000; Jackson et al., 2002), while cognitive aging influences age-related vision dysfunction (Bennett et al., 1999; Hedden and Gabrieli, 2005; Loerch et al., 2008), and contrast sensitivity and spatial learning are affected by aging (Faubert, 2002; Betts et al., 2007; Yamamoto and Degirolamo, 2012).

Visual information from the binocular retina is transmitted to the primary visual cortex, responsible for visual perception of objects’ movement, size, and color, which is located in the occipital lobe. The visual information stream is then transmitted to other cortex for stereopsis, depth perception, and other advanced visual function. Neurons in the primary visual cortex are responsible for orientation, while middle temporal (MT) functions for motion direction of objects (Gold and Ding, 2013; Patten et al., 2017).

During psychometric tasks, choosing between two alternative forced choices is a common method for vision quantification, where participants rely on a neural decoding process to select an option (Gold and Ding, 2013). During the judgment process of the orientation or motion direction, neurons in the visual cortex responsible for the same orientation are activated, while those responsible for the opposite orientation are suppressed (Wang, 2008; Gold and Ding, 2013). The cortex integrates different types of neuronal signals and produces a decision only when the integral signal reaches a threshold. Hence, reduced neuronal inhibition contributes to false selections in orientation and motion direction discrimination tasks, which are thought to be a marker of aging. However, most previous alternative forced-choice psychophysics tasks are coarse with two opposite directions of motion (Snowden and Kavanagh, 2006; Allen et al., 2010), while fine tasks that require participants to discriminate between two similar directions of motion are rare (Ball and Sekuler, 1986; Pilz et al., 2017).

Although previous studies have shown that orientation sensitivity decreases with age in human and macaque monkeys (Schmolesky et al., 2000; Betts et al., 2007; Govenlock et al., 2009; McKendrick et al., 2018), the perceptual orientation tuning curve did not significantly change in aged participants when using a sine-wave raster in various orientations, despite significant differences in the contrast sensitivity between the young and aged groups were observed (Delahunt et al., 2008). To date, the effects of aging on the visual sensory system remain unclear.

In this study, we used MATLAB software to create two different types of new tasks to determine the visual sensory ability, including orientation and motion direction discrimination, during aging. In both tasks, the discrimination threshold, bias of the perceived midpoint, and lapsing rate in participants of different ages were recorded and calculated based on a previous method (Tzvetanov, 2012). For the orientation tasks, we measured discrimination ability using Gabor patches placed at different angles, while for motion direction tasks, we measured discrimination capacity using several random dots moving in different directions. Our new tasks are suitable for a broader age range and could be used to determine potential visual perception alterations in aging (Casco et al., 2017; Pilz et al., 2017).

## Materials and Methods

### Participants

A total of 57 healthy adults aged 22–72 years were recruited through annual physical examinations and were divided into two groups: young [*n* = 28, age (mean ± SEM): 29.04 ± 1.40 years] and old (*n* = 28, age: 57.29 ± 1.08 years) groups. All participants underwent a complete binocular health examination by an experienced ophthalmologist for visual diseases, such as strabismus, amblyopia, cataract, and macular degeneration. Participants’ binocular best-corrected visual acuity was not greater than 0.2 logMAR, and all had participated with their best-corrected visual acuity. Participants with the following conditions were excluded: strabismus, anisometropia, diabetes mellitus, history of eye pathology or operations, or central system diseases. All participants were instructed and trained on similar tasks to familiarize themselves with the tasks and the definition of left and right of stimuli before formal testing. This study was approved by the local ethics committee.

### Stimulus

All participants were tested in a dark room, where the screen was the only light source. A 21-inch CRT monitor (Sony Corporation, Japan) was used as the displayer for all tasks, which has 1,024 × 768 pixel resolution with an 85-Hz refresh rate. The environment was silent and tidy, with only a participant and a researcher in the room. The monitor screen was turned on 30 min before the tasks for stabilization. The screen luminance was calibrated to control display contrast. Stimuli were generated using MATLAB Version R2016a with the Psychophysics toolbox (Brainard, 1997; Pelli, 1997). A chin forehead rest was used to stabilize the eyes at 1,500 mm in front of the screen.

**FIGURE 1:**
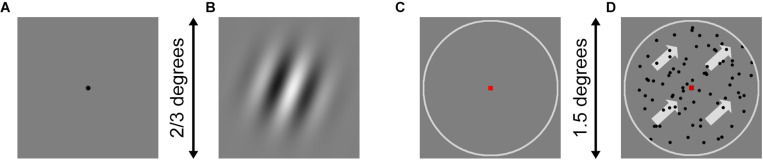
Example stimuli for orientation and motion direction tasks. **(A)** The fixation dot for orientation was presented at first while waiting for the participants to prepare. **(B)** When participants pressed the space button, the fixation dot disappeared, and the Gabor patches were presented for 150 ms. The spatial frequency of the Gabor patch was 3 cyc/deg and filtered with a gradual Gaussian blur from the center to the edge and a circle mask. The visual angle of the Gabor patch was 2/3° when participants sit 1,500 mm in front of the screen. **(C)** The fixation square and circle for motion direction were presented at first while waiting for the participants to prepare. **(D)** When the participants pressed the space button, the moving dots moved in the same direction for 200 ms. The visual angle of the white circle was 1.5° for the participants when 1,500 mm in front of the screen.

In the orientation discrimination task, a Gabor patch was produced, which illustrated a circular sine-wave raster without background noise and had a gradual Gaussian blur edge. The frequency and contrast of Gabor patches were constant, while the spatial frequency of the Gabor patch was 3 cyc/deg. The Gabor patch was presented at different angles relative to the vertical axis for 150 ms in each trial. Representative stimuli are shown in Figures 1A,B. In the motion direction discrimination task, 80 random dots were randomly allocated to the center circle area of the displayer. All the dots moved in the same direction inside the circle. If a dot moved out of the circle, it disappeared and reappeared at the opposite edge of the circle to continue moving. The dots were presented for 200 ms in each trial. The representative stimulus is shown in Figures 1C,D.

### Procedure

**FIGURE 2:**
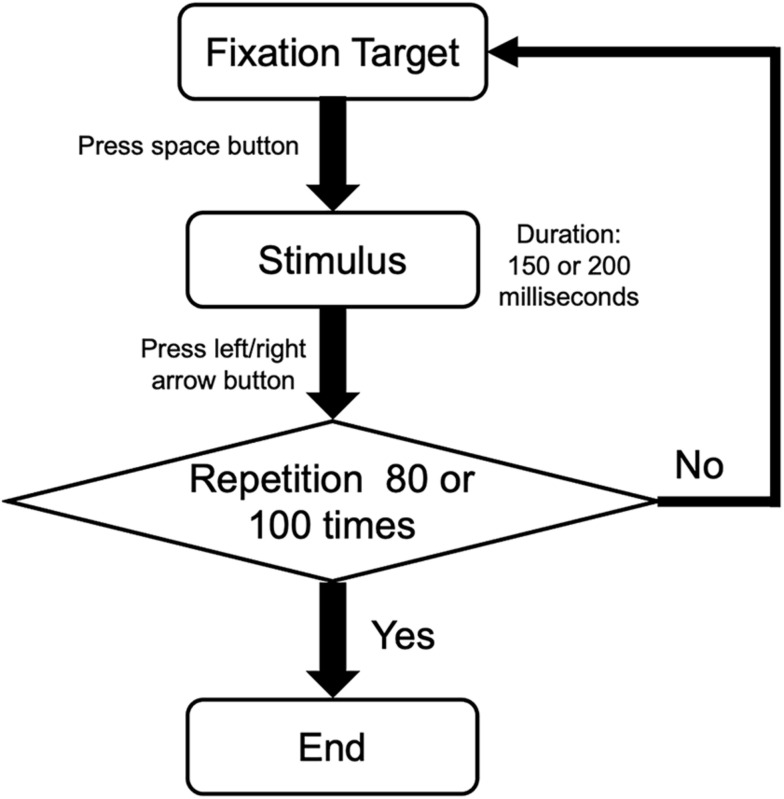
The procedure of a single task in orientation and motion directions. Participants performed a two-alternative forced-choice task. After pressing the space button, participants pressed the left or right button according to the orientation of Gabor patches or the motion direction of random dots. Then, the procedure was repeated 80 times for orientation task and 100 times for motion direction task.

During the experiment, the participants completed an orientation discrimination task and a motion direction discrimination task independently. All responses were inputted using a standard keyboard. In each trial, participants were asked to stare at the center of the black dot/red square of the screen binocularly and to press the space button to present the stimulus and the left/right button to submit responses. For the orientation task, participants were required to indicate the orientation of the static Gabor patch (counterclockwise deviation versus clockwise deviation from the vertical). For the motion direction task, participants were required to indicate the direction of the moving dots (counterclockwise deviation versus clockwise deviation from the upward). There were 100 trials in the orientation task and 80 in the motion direction task. The repeating procedure is illustrated in Figure 2.

The psychophysical task measurements were performed using a weighted up–down adaptive method to improve the efficiency and reliability of the task (Treutwein and Strasburger, 1999). The orientation task included two staircases that started from -21° and +21°, respectively. The presented stimuli were alternately from the staircases. The two staircases were assigned up/down steps of 5/2° and 2/5°, with convergence points of 71.43 and 28.57%, respectively. In both staircases, the next stimulus would increase the number of steps if the current answer were correct, and *vice versa*. Each staircase consisted of 50 trials. Representative psychometric curves are shown in Figures 4A,B. The motion direction task also included two staircases that started at -15° and +15°, respectively. The presented stimuli were from the staircases alternately. The two staircases were assigned up/down steps of 3/1° and 1/3°, with convergence points of 75 and 25%, respectively. Each staircase consisted of 40 trials. Representative psychometric curves are shown in Figures 6A,B. No feedback on the answer correctness was provided to the participants.

### Data Analysis

**FIGURE 3:**
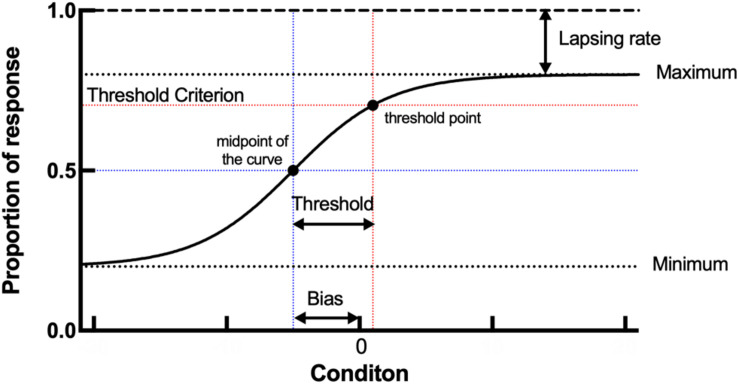
Example plots of a young participant and an old participant showing the raw data analysis in the orientation task. (**A,B**, respectively) Examples of the staircase plots showing the within-staircase trail number *versus* the orientation angles in Gabor patches for a young participant and an old participant. Open symbols are for convergence point of 28.57% and filled symbols for convergence points of 71.43%. The negative angles represent counterclockwise relative to the vertical, and *vice versa*. **(C)** Examples of the plots showing the proportion of the clockwise responses *versus* the orientation angles in Gabor patches and the respective psychometric functions. The black X shape, rhombus, and curve (function parameters: *l* = 0.0072, *μ* = 1.5518, and *σ* = 2.4718) for an old participant. The blue dots, rhombus, and curve for a young participant (function parameters: *l* = 0.0028, *μ* = 0.0782, and *σ* = 0.9106). Rhombus is the midpoint of the curve.

All participants’ responses to the respective stimulus degrees were analyzed, as described previously (27, 31). Briefly, for each task, data were analyzed by calculating the proportion of clockwise responses at each deviation degree *x*: *p_*x*_ = y_*x*_ /n_*x*_*. For the orientation task, *y*_*x*_ is the count of clockwise responses from the vertical direction at the deviation degree *x* (or the upward motion direction for the motion direction task), and *n*_*x*_ is the total number of trails at the deviation degree *x*. For degree *x*, a negative value indicates counterclockwise, while a positive value indicates clockwise. This experimental dataset {*x*_*i*_,*p*_*i*_} is modeled as a logistic function:

$$p\,\left(x\right)=l+\frac{1-2\,l}{1+\text{exp}\,\left(-\frac{\ln\left(21/4\right)}{\sigma }\times \left(x-\mu \right)\right)}$$

where *l* is the lapsing rate of the participant, *μ* is the perceived midpoint or the reference point of the participant, and *σ* is the discrimination threshold around the midpoint. This function was adjusted based on the data using Bayesian fitting (Treutwein and Strasburger, 1999). In this study, we extracted the threshold, bias, and lapsing rate of each participant in the orientation and motion direction tasks. The threshold (equal to *σ*) refers to the minimum angles from the vertical or upward motion direction; the bias (equal to the absolute value of *μ*) represents the difference between the perceived participant’s midpoint and the vertical or upward motion direction; the lapsing rate (i.e., 1–the maximum of the logistic function) indicates that of the participant. To better understand the parameters, we constructed a diagram to show an example of psychometric curve and the meaning of the threshold, bias, and lapsing rate (Figure 3).

To assess discrimination ability differences in orientation and motion direction tasks between the young and old groups, GraphPad Prism 9.0 (GraphPad Software, United States) was used to perform the Mann–Whitney test for abnormal distribution data. For correlation analysis of age and threshold, bias, and lapsing rate, Spearman’s rank correlation was performed using GraphPad Prism 9.0. A *p*-value of <0.05 was considered statistically significant.

## Results

### Clinical Details of Participants

All participants enrolled in this study were healthy adults. A total of 56 participants performed the assigned two tasks. Before the experiment, all participants underwent thorough physical and eye examinations. Table 1 summarizes the clinical information of participants.

**TABLE 1:** Clinical information of both groups, including age, sex, best-corrected visual acuity, and education background.

Clinical details	Young group	Old group
Number	28	28
**Age, years**		
Mean ± SD	29.04 ± 7.42	57.29 ± 5.72
Median, *n* (range)	25.5 (22–49)	56 (50–72)
**Sex**		
Female	21	18
Male	7	10
**Best corrected visual acuity, logMAR**		
OD, Mean ± SD	0.025 ± 0.065	0.046 ± 0.074
OS, Mean ± SD	0.025 ± 0.075	0.025 ± 0.070
**Education background**		
Primary school	2	10
Middle school	2	17
College and above	24	1

### Orientation Discrimination

**FIGURE 4:**
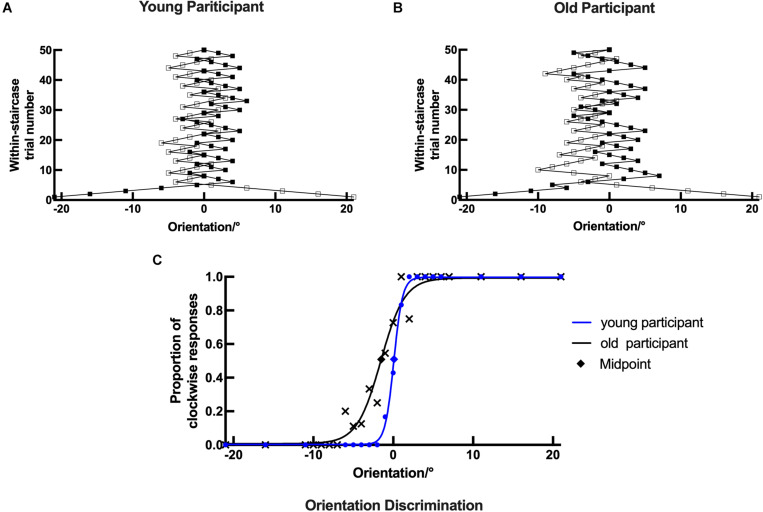
The discrimination ability difference of orientation between the young and old groups. **(A)** Statistical results showing the threshold differences between the young (*n* = 26) and old (*n* = 26) groups. **(B)** Statistical results showing the bias differences between the young (*n* = 28) and old (*n* = 27) groups. **(C)** Statistical results showing the lapsing rate differences between the young (*n* = 28) and old (*n* = 25) groups. Data are expressed as mean ± SEM. *p < 0.05 for the young group and the old group in the threshold and bias. ns indicates no significant difference between the young and old group in the lapsing rate analysis.

In the orientation discrimination experiment, both young and old participants were required to indicate the orientation of the static Gabor patch (left versus right). Each response was recorded as staircase plots according to the convergence point. Example plots are shown for a young participant and an old participant in Figures 4A,B, respectively. For the young participant, the staircase was straight to the midpoint and formed a zigzag around the vertical direction (0°), while for the old participant, the staircase plots were wider and repeated irregularly, suggesting that the old participant could not discriminate the small angles clearly in the experiment.

**FIGURE 5:**
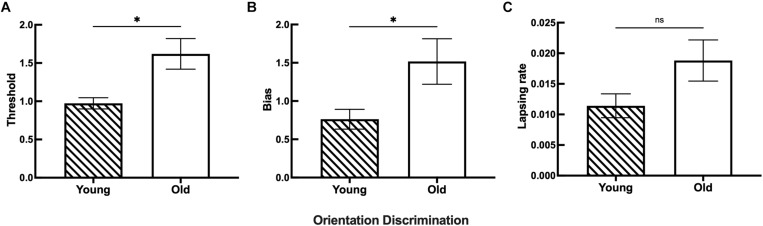
A diagram of psychometric curve and meaning of threshold, bias, and lapsing rate. The threshold represents the horizontal distance between the threshold point and midpoint of the curve. The bias represents the horizontal distance between the midpoint and *x* = 0. The lapsing rate represents the vertical distance between the maximum of the curve and *y* = 1.

**FIGURE 6:**
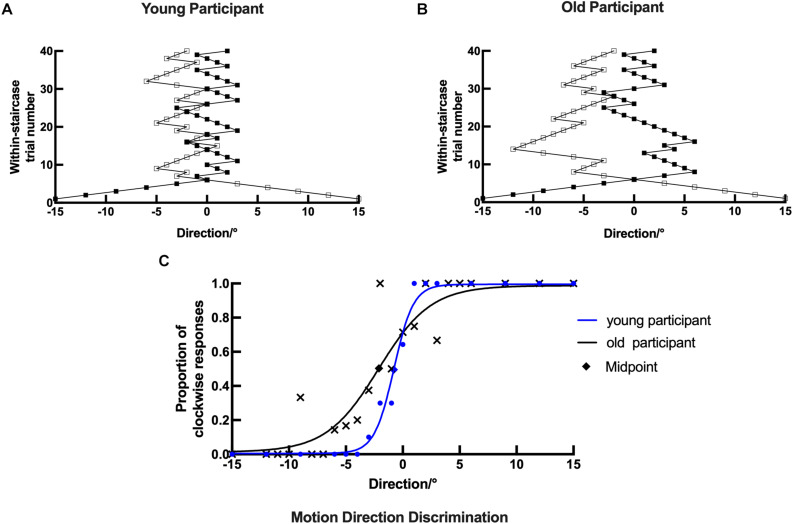
Example plots of a young participant and an old participant showing the raw data analysis in the motion direction task. (**A,B**, respectively) Examples of the staircase plots showing the within-staircase trail number *versus* the motion direction angles for a young participant and an old participant. Open symbols are for convergence point of 25%, and filled symbols are for convergence points of 75%. The negative angles represent counterclockwise relative to the upward motion direction, and *vice versa*. **(C)** Examples of the plots showing the proportion of the clockwise responses *versus* the angles in motion direction and the respective psychometric function. The black X, rhombus shape, and curve (function parameters: *l* = 0.0124, *μ* = 2.1248, and *σ* = 3.8050) for an old participant. The blue dots, rhombus, and curve for a young participant (function parameters: *l* = 0.0049, *μ* = 0.7850, and *σ* = 0.4270). Rhombus is the midpoint of the curve.

Example plots are shown for a young participant with the following parameters: *l* = 0.0028, *μ* = 0.0782, and *σ* = 0.9106 and an old participant with the following parameters: *l* = 0.0072, *μ* = 1.5518, and *σ* = 2.4718 in Figure 4C. The threshold, bias, and lapsing rate of all participants were used for further statistical analysis. The shapes of psychometric curves vary in slope, horizontal shift, and height with different parameters, which can be used to compare the performance between young and old participants directly. Compared with the old participant, the young participant’s datasets were nearly located on the respective curve having a larger slope and height and a smaller horizontal shift (Figure 4C). These results suggest that old participants had decreased ability and increased error rate in the discrimination of fine orientation difference.

The results for both young and old participants are summarized in Figure 5. Compared with the young group, the old group had a higher threshold of orientation discrimination (Figure 5A), an increased bias of the perceived midpoint of orientation (Figure 5B), and a larger lapsing rate in the orientation task (Figure 5C); the differences in the threshold and bias between the two groups were significant. This result suggests that in fine orientation discrimination, aging participants need stronger stimuli to discriminate clearly. In addition, the vertical orientation judged by aging participants was more oblique, suggesting an imbalance of neural mechanisms specialized for orientation discrimination in aging.

### Motion Direction Discrimination

In the motion direction discrimination experiment, both young and old participants were required to indicate the motion direction of the dots (leftward versus rightward). Each response was recorded as staircase plots based on the convergence point. Example plots are shown for a young participant and an old participant in Figures 6A,B, respectively. Similarly, for the young participant, the staircase was straight to the midpoint and formed a zigzag around the upward motion direction (0°). However, the staircase plots in the old participant were wider and repeated irregularly, suggesting that the old participant could not discriminate moving dots with small motion direction difference well in the experiment.

The data analysis process in the motion direction discrimination task was similar to that in the orientation discrimination task. Example plots are shown for a young participant with the following parameters: *l* = 0.0049, *μ* = 0.7850, and *σ* = 0.4270 and an old participant with the following parameters: *l* = 0.0124, *μ* = 2.1248, and *σ* = 3.8050 in Figure 6C. The psychometrics curve difference between young and old participants can also be used to compare their performance directly. Compared with the old participant, the young participant’s datasets were nearly located on the respective curve, and the curve had a larger slope and height and a smaller horizontal shift (Figure 6C). These results suggest that old participants had impaired ability in discriminating stimuli with fine motion directions.

**FIGURE 7:**
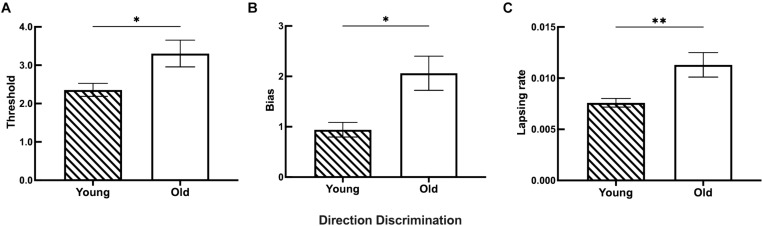
The discrimination ability difference of motion direction between the young and old groups. **(A)** Statistical results showing the threshold differences between the young (*n* = 26) and old (*n* = 27) groups. **(B)** Statistical results showing the bias differences between the young (*n* = 23) and old (*n* = 26) groups. **(C)** Statistical results showing the lapsing rate differences between the young (*n* = 27) and old (*n* = 24) groups. Data are expressed as mean ± SEM. *p < 0.05 for the young group and the old group in the threshold and bias analyses. **p < 0.01 for the young group and the old group in the lapsing rate analysis.

The results for both young and old participants are summarized in Figure 7. Older participants had a higher threshold of motion direction discrimination than young participants (Figure 7A). In addition, old participants had an increased bias of the perceived midpoint in motion direction discrimination (Figure 7B), and significantly higher lapsing rate than that of young participants (Figure 7C).

## Discussion

In this study, we used two new tasks to investigate changes in fine visual perception in a normal aging population and compared the discrimination ability of micro-angle changes between young and old participants. The results showed that (1) compared with young adults, aging adults had a higher threshold, an increased bias, and a higher lapsing rate in the orientation task, although some differences were not statistically significant; (2) in the motion direction task, old participants had a higher threshold, an increased bias, and a significantly higher lapsing rate than young participants. These findings suggest that old adults have an altered visual perception. We also analyzed the correlation of age and threshold, bias, and lapsing rate in orientation and motion direction discrimination tasks. In the orientation discrimination task, the log threshold was significantly correlated with age (regression coefficient *r* = 0.3199, *p* < 0.05); the log bias (regression coefficient *r* = 0.2386, *p* = 0.0793) and log lapsing rate (regression coefficient *r* = 0.1374, *p* = 0.3267) showed an increased trend with age, although not significant (Supplementary Figure 1). In the motion direction task, the log threshold (regression coefficient *r* = 0.3221, *p* < 0.05), log bias (regression coefficient *r* = 0.3365, *p* < 0.05), and log lapsing rate (regression coefficient *r* = 0.3698, *p* < 0.01) were all correlated with age (Supplementary Figure 2). However, the increasing trend of these parameters might be not accordant with different age ranges. A previous study has reported piecewise linear functions between age and contrast sensitivity (Tang and Zhou, 2009), while another has also showed similar sensitivity to motion in participants with lower age ranges (Bennett et al., 2007). This might be attributed to the stable discrimination ability in young adults. Only when the visual function impaired beyond compensatory ability with aging, the abnormal discrimination ability could be detected.

In the orientation discrimination tasks, a higher threshold among aged participants indicated that old individuals might have worse orientation discrimination ability of small angle changes, although their visual acuity might be normal. The bias of the aged participants was increased, suggesting an imbalance of neurons responsible for different orientations in the visual cortex. In addition, old participants had a relatively high lapsing rate in the orientation task, partially due to a limited number of participants and the fact that the static sine wave raster tasks are relatively easy for aging individuals compared with the motion direction task.

In the motion direction discrimination tasks, old participants required stronger stimuli to make a correct judgment, suggesting a decreased discrimination ability of motion dots. Furthermore, participants in both groups had a higher threshold in motion direction tasks than in orientation tasks, suggesting a higher sensitivity of the motion direction task in aging. This result may be explained by the different visual process streams in the visual cortex for the two stimuli. Meanwhile, receiving visual information is an “encoding” procedure to extract motion features, while discrimination is the “decoding” procedure to make a judgment regarding the moving direction (Graham, 1989). This complex task involving multiple visual areas could show clearer vision perception changes in healthy aging.

In addition, previous studies have found that the discrimination ability difference of motion direction tasks between young and old participants increased when the dots moved at lower speeds (Snowden and Kavanagh, 2006). In this study, the stimuli dots moved at 8°/s, similar to that in a previous study, and our results are in line with that of Snowden and Kavanagh (2006). Moreover, the effect of stimulus size on discrimination remains controversial. When the stimulus size increases, the number of dots also increases to maintain the density; the increased size may activate the cortex responsible for both the central and peripheral visual fields; reduced peripheral suppression was reported in older participants (Mapstone et al., 2008; Betts et al., 2009). Meanwhile, the number of dots can influence discrimination ability (Betts et al., 2005). In our study, the stimulus size was only 1.5°, a small central circle in the vision field, which was associated with the central visual field and less affected by the surrounding area.

Both anatomy and neurophysiology studies on aging have shown that impaired visual perception might be correlated with brain deterioration in older population. Myelinated fibers and synapses in the brain regions, including the V1 area, are degraded in aging monkeys (Peters et al., 2001; Peters, 2009). These anatomical changes may be partially responsible for the increased response time and decreased vision perception. In this study, higher lapsing rate and increased bias of older participants were observed in the motion direction tasks, which is in line with previous findings (Wang et al., 2005).

In addition to structural changes in aging brains, decreased selectivity and increased spontaneous brain activity have also been demonstrated in senescent mammals (Delahunt et al., 2008). Studies have suggested that orientation and direction selectivity are reduced in aging monkeys and cats (Wang et al., 2006; Yu et al., 2006), and that acetylcholine and GABAergic neurotransmitters are important for orientation selectivity, indicating that insufficient inhibition of cortical neurons may contribute to age-related vision impairment (Schmolesky et al., 2000; Andersen and Ni, 2008; Edden et al., 2009; McKendrick et al., 2018). In addition, it has also been reported that reduction of orientation selectivity is reversed by local administration of GABA agonists (Leventhal et al., 2003), and downregulation of inhibitory neurotransmitters in aging contributes to hyperactivity of the visual cortex (Delahunt et al., 2008). A previous study has also found that orientation detection thresholds were significantly negatively correlated with visual cortex GABA levels for obliquely oriented patterns, and gamma oscillation frequency was positively correlated with GABA levels in the primary visual cortex, suggesting that visual perception is impaired in the healthy aging population (Edden et al., 2009). In this study, the bias of old participants in both tasks was nearly double than that of the young participants, which may partially be due to the GABAergic neurotransmitter level change or the increased brain activity and background noise in the visual cortex.

A contrast, which is the basis of visual perception, is the difference between light and dark conditions. In macaque brains, the primary visual cortex (V1) is involved in contrast perception (Ruiz and Paradiso, 2012). V1 activity is also associated with the contrast stimuli (Niemeyer and Paradiso, 2017). Studies have shown that the contrast of a target could influence the discrimination ability of healthy participants, especially aging participants (Allen et al., 2010). In this study, the orientation discrimination tasks were dependent on the contrast sensitivity and spatial frequency selectivity to excite orientation-tuned neurons and recognize Gabor patches. As the contrast decreases, the accuracy also decreases, and participants’ discrimination becomes less reliable with increased variability (Tzvetanov, 2012). Hence, in this study, the target contrast was 0.9 in the orientation tasks and 1.0 in motion direction tasks to ensure reliability and efficiency. Our results illustrated the discrimination ability changes of small angles among older participants, even with sufficient contrast stimuli.

Notably, the motion direction task primarily evaluates the visual cortex neurons’ ability to process moving objects. The analysis mechanism of motion direction in visual cortex differs from that of static contrast stimuli. Different neurons in the visual cortex can be activated by different directions of stimuli; this phenomenon is referred to as direction selectivity (Tzvetanov, 2012; Chaplin et al., 2018). However, only a small number of neurons in the V1 area exhibit direction selectivity, although visual information from both eyes is transmitted to V1 at the first stage in primates (Davies et al., 2016). Most neurons in the MT show a strong directional selectivity, which might be associated with the motion direction discrimination task (Albright, 1984; Elston and Rosa, 1997). An fMRI study reported functional changes in the MT area in response to stimuli of drifting Gabor patches in different sizes and contrasts, suggesting that the MT area might be involved in the direction-selectivity process (Turkozer et al., 2016). In this study, the motion direction tasks adopted a random-dot pattern without orientation cues. Based on our findings and those of previous studies, the decreased discrimination of motion direction may demonstrate the aging effect on the visual cortex, especially in the MT area. Meanwhile, the increased bias in the motion direction task may suggest an imbalance of direction-selectivity neurons in aging.

## Conclusion

In this study, we have developed two new psychometric tasks to investigate fine orientation and motion direction discrimination in young and old adults with normal visual acuity. Our results illustrated that aging participants exhibited increased threshold and bias in both tasks, and a higher lapsing rate in the motion direction task. Compared with previous orientation and motion direction tasks, our tasks are more rapid and simpler for fine visual perception in older individuals (Pilz et al., 2017). Although visual acuity remains to be examined clinically, other psychometric examinations or treatment methods need to be developed to prevent or delay visual perception in aging.

## Data Availability Statement

The raw data supporting the conclusions of this article will be made available by the authors, without undue reservation.

## Ethics Statement

The studies involving human participants were reviewed and approved by the Ethics Committee of The First Affiliated Hospital of Anhui Medical University. The patients/participants provided their written informed consent to participate in this study.

## Author Contributions

LF designed the research. LX, JD, and SL performed the tasks for subjects. HC wrote the Matlab programs. LX and HC wrote the article. All authors contributed to the article and approved the submitted version.

## Conflict of Interest

The authors declare that the research was conducted in the absence of any commercial or financial relationships that could be construed as a potential conflict of interest.
