# Supplementary material for: Decline of Orientation and Direction Sensitivity in the Aging Population
Source: Front Neurosci. 2021 Apr 7;15:643414. doi: 10.3389/fnins.2021.643414 (PMC8064032; doi:10.3389/fnins.2021.643414)
Supplement: Supplementary file 1 [file Data_Sheet_1.docx]

Supplementary Material

**Data Availability Statement**

The raw data supporting the conclusions of this article will be made available by the authors, without undue reservation.

**Supplementary materials**

The discrimination ability of orientation and motion direction is different in young and old participants. We also analyzed the relationship of log threshold, log bias and log lapsing rate in two tasks with age. In orientation discrimination task, the log threshold was significantly correlated with age (regression coefficient r = 0.3199, *p* < 0.05); The log bias (regression coefficient r = 0.2386, *p* = 0.0793) and log lapsing rate (regression coefficient r = 0.1374, *p* = 0.3267) showed an increased trend with age without statistical significance (Supplementary Fig.1). In the motion direction task, the log threshold (regression coefficient r = 0.3221, *p* < 0.05), log bias (regression coefficient r = 0.3365, *p* < 0.05), and log lapsing rate (regression coefficient r = 0.3698, *p* < 0.01) were correlated with age (Supplementary Fig. 2).


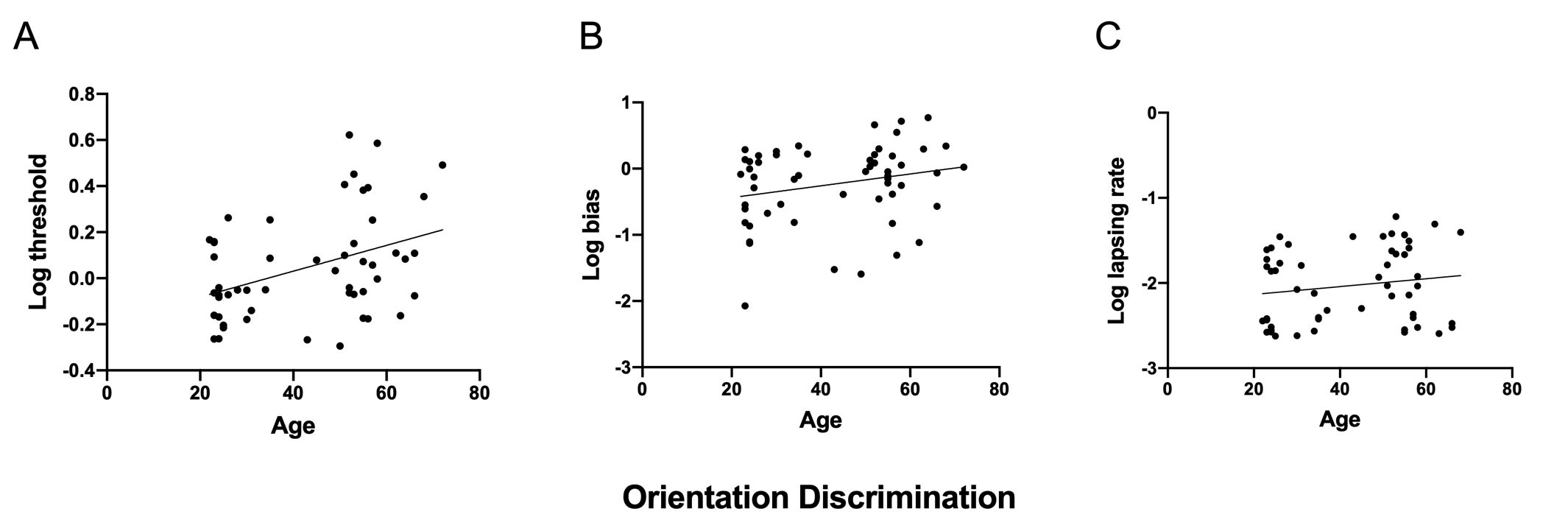


**Supplementary Figure 1.** Correlations between age and log threshold (A), bias (B), and lapsing rate (C) respectively in orientation discrimination tasks.


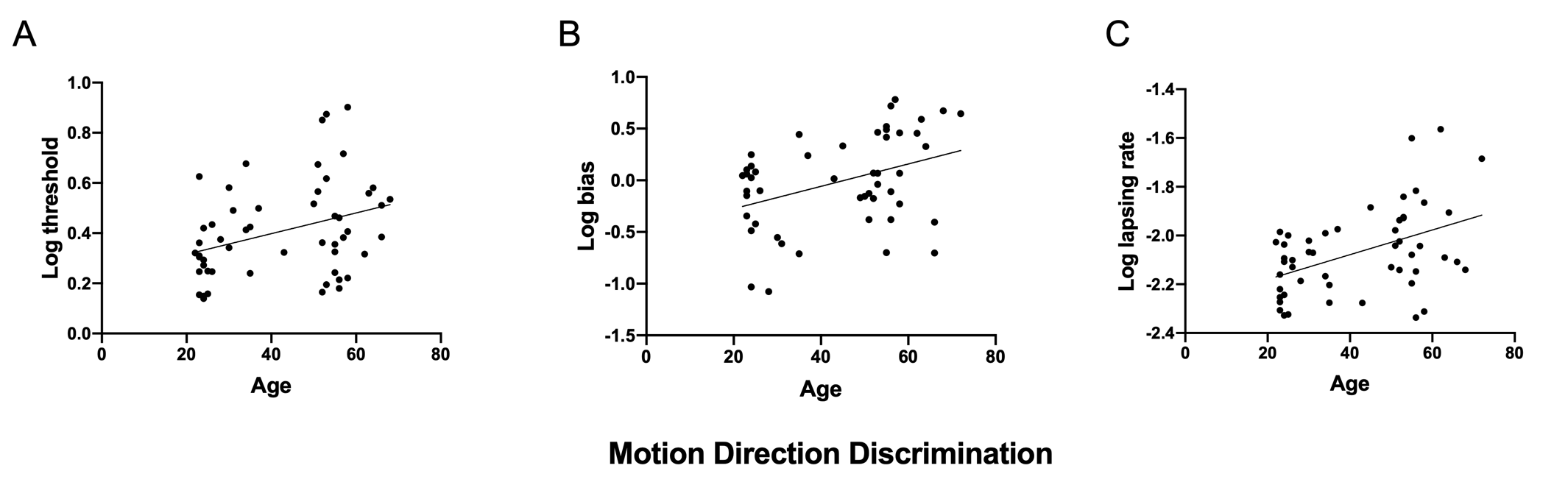


**Supplementary Figure 2.** Correlations between age and log threshold (A), bias (B), and lapsing rate (C) respectively in motion direction discrimination tasks.
